# Reappraising a Parent can Occur With Non-suggestive Questions: Changing Emotions and Memories of Emotion

**DOI:** 10.1177/00332941241283413

**Published:** 2024-09-11

**Authors:** Lawrence Patihis, Mario E. Herrera

**Affiliations:** Department of Psychology, 6697 University of Portsmouth, Portsmouth, UK; Department of Psychology, 8620 University of North Carolina, Asheville, NC, USA

**Keywords:** Reappraisal, mother, parent, emotion, memory of emotion

## Abstract

Whether it is possible to reappraise parents using non-suggestive questions, and whether this has an impact on emotions and memories, is of great interest in both life and psychotherapy. Past research has shown reappraisals of past situations is associated with changes in memories of emotions. In previous work we showed memories of love could be affected by reappraisals, but did not analyze that dataset on other memories of emotion. The current paper investigates the effect of reappraisals toward participants’ mothers on the emotions: happiness, interest, sadness, and anger (and on memories of those emotions in childhood). Results show that emotions appeared to be significantly changed by reappraisals. In Experiment 1 (*N =* 301; *M*_age_ = 36), we found memories of emotion were affected, especially memory of happiness in childhood, but to a lesser degree compared to current emotions. This offered some confirmation of the cognitive appraisal view of memories of emotions. Experiment 2 (*N =* 202; *M*_age_ = 36) with pretest and posttest measures showed some similar patterns, but with slightly muted effects. Therapists and clients should be aware that non-suggestive prompts might lead to reappraisals of parents, with knock on effects on emotions and memories. Whether this should be part of informed consent in therapy is open to debate.

## Introduction

Negative reappraisals of parents have been a potential iatrogenic aspect of talk therapy since Freud popularized the analysis of parental relationships (Breuer & Freud, 1895/1953). One concern of such reappraisals is that they might affect emotions and memories of emotions toward a parent. For example, memories of emotions felt toward a parent in childhood may impact the ongoing relationship with the parent. Past research has suggested that memory of emotions for events can change as current cognitions change (Levine, 1997). The explanation of this effect is that as memory fades over time, a person uses current cognitive appraisals of the past when reconstructing their memory. Previous experiments have found that memory of love is impacted by changing appraisals of a parent (Patihis, Cruz et al., 2019). But no previous research has investigated the effect of reappraising a parent on other emotions (such as basic emotions) and memories of those emotions in childhood. The two current experiments, use datasets from Patihis, Cruz et al. (2019); using variables not previously analyzed) to explore whether manipulating current appraisals in adult participants changes current emotions toward their mother (happiness, interest, sadness, anger), and also bias childhood memory of emotions.

Memory is reconstructive and malleable in nature (e.g., Bartlett, 1932; Katz, 1989; Loftus, 2005), and there is some evidence that this is true of memory of emotions as well. Evidence suggests that memory of emotions appear to change according to current appraisals of an event (Levine, 1997). The overall theory to emerge from such research is that changes in current appraisals of goal relevant aspects of the past situation (or person), can bias the recall of emotions related to that situation (Levine, 1997; or person: Patihis, Cruz et al., 2019). This theory contrasts with some earlier theories that emotions were relatively unchangeable (LeDoux, 1992), and is contrary to the catharsis model of emotion as well (Breuer & Freud, 1893; but see Bushman, 2002). Past research, summarized in Levine et al. (2009), has suggested that changing cognitive appraisals partially cause changes in memories of emotions.

There is some multi-stage research that demonstrates the instability of memory of emotions. Levine (1997) found that supporters of the U.S. presidential candidate Ross Perot tended to bias their memories of emotion about his withdrawal from the race in accordance with changes in their appraisal of him. Similarly, Levine et al. (2001) found that changes in memory of emotions towards the not guilty verdict of O.J. Simpson were found over time in directions consistent with shifting current appraisals (happiness, anger, and surprise). Safer et al. (2001) found evidence to suggest that widows whose current negative emotions about losing a spouse did not diminish much over several years tended to overestimate how much grief they had felt initially. Also in a longitudinal study, Levine et al. (2021) found that memory of emotion about political events was malleable over time. These studies are valuable in that they documented naturally occurring changes in appraisals and memory of emotion, but they did not use a true experimental design (e.g., manipulation and random assignment). We examine experimental research next.

Only a limited number of experiments have manipulated current appraisals with memory of emotion as the outcome measure. Keuler and Safer (1998) found that students who received positive feedback about their exams (that they achieved a good grade) exaggerated their memories of pre-exam anxiety, compared to those that received no feedback. In contrast, Safer et al. (2002) found that after an exam, undergraduates receiving positive feedback about their grade *underestimated* pre-exam anxiety. More recently, Chang and Overall (2024) found that memories of depressive symptoms during a Covid-19 2020 lockdown tended to be biased by current lockdown experiences (in 2021). They also found that with their particular cognitive reappraisal task was associated with remembering more depressive symptoms than participants actually initially reported (in a contrary direction to what they predicted).

In perhaps the most relevant past experimental research to the current study, Patihis, Cruz et al. (2019) found that changing current appraisals that participants held of their mothers seemed to significantly impact memories of love towards their mothers. This particular experiment was distinct from other previous experiments in that it varied the experiment groups by level and direction of cognitive reappraisal (appraisal up; appraisal down; and control condition). Memories of love toward mothers tended to be higher in appraisal up conditions, compared to appraisal down conditions. In summary, experimental and longitudinal research has suggested a possible relationship between changing appraisals and changing memory of emotions.

### The Current Experiments

Past research discussed above has shown cognitive reappraisals may affect memories of emotion. In particular, Patihis, Cruz et al. (2019) found reappraisals affected memories of love, and collected other data on other emotions as well that could not fit into that paper—and are reported in the current paper. The current experiments examine the unexamined data specifically focusing on the emotions happiness, sadness, anger, and interest. These emotions were chosen because some are considered basic emotions (happiness, sadness, anger; interest being the only emotion not usually considered one of the five basic), to examine both negative and positive emotions, and to incorporate a mix of pre-goal and post-goal emotions. The participants were randomly assigned into conditions that either increase current cognitive appraisals of mothers (Mother Appraisal Up condition), to decrease it (Mother Appraisal Down condition), or to serve as control conditions (Null; Teacher Appraisal Down conditions). Based on past research and the argument outlined above, here are the hypotheses:

**Hypothesis 1.** As a manipulation check, the independent variable manipulation (writing prompts) should significantly change current appraisals of participant’s mothers (a measure of our IV).

**Hypothesis 2.** When current appraisals go up, participant’s current feelings of positive emotions felt towards their mother will go up (happiness, interest), and negative emotions will go down (anger, and sadness). The Appraisal Up condition will elicit significantly different scores on current feelings of emotions, compared to the Appraisal Down condition. This hypothesis is predicted from an extension of Lazarus’s (1991) cognitive appraisal theory of emotion.

**Hypothesis 3.** If current appraisals go up, the memories of positive emotions towards mothers will go up, and memories of negative emotions will go down. Specifically, participant’s memories of happiness, interest, anger, and sadness towards their mother from years in childhood (average of first [ages 6-7], sixth [11-12], and ninth [14-15] grade). This hypothesis is supported by past research that has given some indication that reappraisals may change memories of emotions (e.g., Chang & Overall, 2024; Levine, 1997).

## Experiment 1

Experiment 1 used an experimental design with posttest measures of the dependent variables. The four conditions were: Mother Appraisal Up, and Mother Appraisal Down to manipulate current appraisals of the participant’s mother—and two control conditions: Null (no writing prompts) and Teacher Appraisal Down. The latter control group contained the same writing prompts as Mother Appraisal Down, but with the word “mother” replaced with “teacher.” This experiment consisted of a between-subjects experimental manipulation in Session 1 to assess the effect of current appraisals on emotion, and memory of emotions towards the participants’ mother. Follow ups measures of the DVs were taken in Session 2 (2 weeks later) and Session 3 (4 weeks later) assessed how long any effects lasted.

## Experiment 1 Method

### Participants

As in Patihis, Cruz et al. (2019), 301 US participants were recruited via the online survey website Amazon Mechanical Turk sample (AMT, Buhrmester et al., 2011; Gosling et al., 2004) of *M*_age_ 36.2 (*SD* = 11.1; range 18–68), with 76.1% (*n =* 229) female, 23.3% (70) male, and .7% (2) choosing “other (please specify).” Race was self-reported as: 75.4% (227) White, 13.6% (41) Black or African American, 9.3% (28) Asian, 3.0% (9) American Indian or Alaska Native, 2.3% (7) Native Hawaiian or Pacific Islander, and 2.7% (7) chose other. Ethnicity was distributed as: 6.6% (20) Hispanic or Latino, with 91.7% (276) not Hispanic or Latino. Mean self-reported socioeconomic status (SES), on a scale from 1 to 10, was 4.97 (*SD* = 1.7).

### Design

The design was a between groups experiment, with only post-test measures of the dependent variables. The independent variable was the participants’ current appraisal of their mother. The dependent variables are memories of various emotions towards the mother in childhood.

### Materials

#### Writing Prompts Appraisal Experimental Manipulations

Participants were randomly assigned to one of four groups. The four experimental conditions’ materials are given in Appendix A, and is summarized below.

##### Mother Appraisal Up condition

Participants in the Mother Appraisal Up condition were administered five writing prompts, and asked to write several sentences to each giving recent examples of when their mother had exhibited evidence of having a positive attribute. These attributes were related in some way to how the parent might facilitate the needs or goals of participants (e.g., competence, generosity, support). The following was one example writing prompt: “Please write 3-4 sentences giving the most recent examples of when your mother showed warmth towards you.” Another example was: “Please write 3-4 sentences giving the most recent examples of when your mother showed competence (effectiveness) in her life.” Other prompts asked for 2-3 sentences of recent examples of their mother showing generosity, good guidance towards the participant, and examples of when the mother gave and received love.

##### Mother Appraisal Down condition

In this condition, participants were administered five writing prompts similar to the Mother Appraisal Up condition prompts. However, participant had to write recent examples when their mother displayed a “lack of” the positive attributes. For example, one writing prompt read: “Please write 3-4 sentences giving the most recent examples of when your mother showed a lack of warmth towards you.” Another example was: “Please write 3-4 sentences giving the most recent examples of when your mother showed a lack of competence (effectiveness) in her life.” Other writing prompts asked for recent examples of the mother showing a lack of generosity, giving bad guidance, and not giving love to the participant.

##### Teacher Appraisal Down condition

In this condition participants were administered writing prompts identical to the Mother Appraisal Down condition, except the word “mother” was replaced by the word “teacher” throughout. To make the prompts fit to this different target-person, the words “not give love to you” from the Mother Appraisal Down condition were changed to “not give praise to you” in the teacher version of the prompts.

##### Null Condition

Those in the control condition received no writing prompts and instead received an arrow at the bottom to proceed to the next page.

#### Current Appraisal of Mothers: Manipulation Check Questions

Participants were presented with a five item scale that asked them to currently evaluate their mother on positive attributes (emotional warmth, generosity, competence, good guidance, and giving and receiving love; see Appendix B for materials). Participants were asked “How do you evaluate your mother *currently* on:” followed by the five items. Two example items were: “Current warmth of your mother” and “Current generosity of your mother.” Beneath each item was a fully anchored Likert-type scale (choices of *1 = poor, 2 = fair, 3 = good, 4 = very good, or 5 = excellent)*. An additional choice of N/A was given, which if chosen was set to give missing data. The mean composite of these five items was taken to form our measurement for current appraisal of mother. The five items yielded a high Cronbach’s α (e.g., Experiment 1, Session 1, Cronbach’s α = .941).

#### Memory of Emotions toward Mother

**Participants were administered an** adapted version of the Memory of Love toward Parents Questionnaire (MLPQ; Patihis et al., 2020; Patihis, Jackson, et al., 2019). This adapted MLPQ focused on the target emotions of happiness, interest, sadness, and anger (see Appendix C for materials). The four subscales presented asked the strength of the memories of the four emotions about the time periods first grade (ages 6-7), sixth grade (ages 11-12), ninth grade (ages 14-15), and current feelings. An example of an item was: “During the whole year when you were in first grade, *how strong on average* was your **happiness** toward your **mother**?” For these questions, the Likert-like scale ranged from *0* = *Nonexistent* to *6 = Extremely Strong.* Other words also used in the questionnaire in substitution for the word “happiness” were “interest”, “sadness,” and “anger.” In all the sessions reported in this manuscript, we also administered memory of love questions (see Patihis et al., 2020) and for reasons of space, focus, and clarity, we reported those results elsewhere (Patihis, Cruz et al., 2019).

#### Positive and Negative Affect Schedule (PANAS)

The PANAS consists of two 10-item subscales representing negative and positive current affect/mood (Watson et al., 1988). In the current experiment, both subscales had high internal reliability (Experiment 1: time 1: positive affect α = .908; negative affect α = .938) and the two subscales were negatively (not significantly) correlated with a small effect size, *r* = −.112, *p* = .052.

### Procedure

Participants in the US signed up on the AMT research website, and participated in our study in return for payment. Demographic questions and parental background questions were administered first. Then, participants were randomly assigned into one of four writing-prompt conditions to manipulate current appraisals of the participant’s mother (Mother Appraisal Up, Mother Appraisal Down; and two control groups: Null, Teacher Appraisal Down). For the wording of the questions and materials see Appendix A. After this experimental assignment, all participants completed questions assessing current appraisal of their mothers (Appendix B). Then participants completed the PANAS, followed by the items on memory of emotion and current emotions towards their mother (Appendix C). After completing this, participants were automatically given a unique code for payment of $1 on AMT. This experiment took approximately 19 minutes. The datasets for both experiments can be found here: https://osf.io/rhfuw/

## Experiment 1 Results

### Hypothesis 1: Manipulation Check

In an ANOVA with all four conditions included as the predictor variable, the Mother Appraisal Down condition (*M* = 2.87, *SD* = 1.32) scored significantly lower on current appraisals of mother compared to the Null (*M* = 3.67, *SD* = 1.08, *p* < .001), Teacher Appraisal Down (*M* = 3.77, *SD* = 1.09; *p* < .001) and Mother Appraisal Up (*M* = 4.11, *SD* = 0.96; *p* < .001) conditions. The Mother Appraisal Up condition scored significantly higher than the Null condition (*p* = .018), but not significantly higher than the Teacher Appraisal Down condition (*p* = .063). Thus, these manipulation checks established the conditions appeared to be successfully manipulating current appraisals—especially when comparing the Mother Appraisal Up condition to the Mother Appraisal Down condition.

### Hypothesis 2: Current Emotions

**Figure 1.**
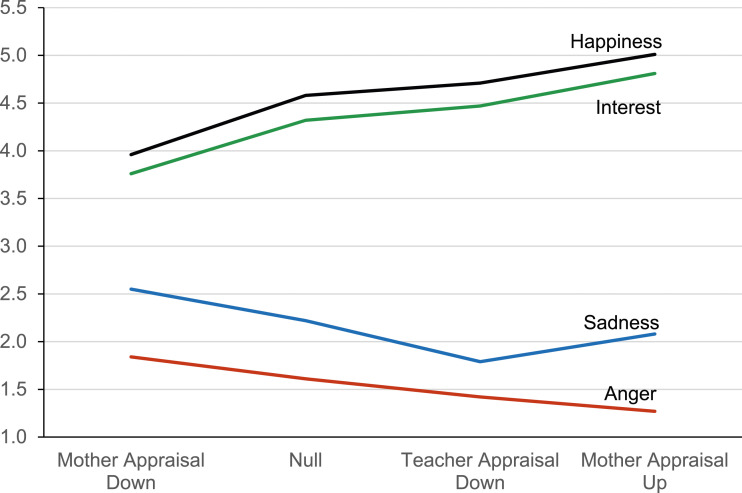
Experiment 1 results showing current emotion towards mothers (*y*-axis) compared by the four experimental conditions (*x*-axis). Notice the general pattern of positive current emotions having an upward slope as one moves from the lower appraisal to the higher appraisal condition, and negative emotions with a more downward slope.

Table 1 displays the means and standard deviations, by condition, of the reported current feelings of happiness, interest, sadness, and anger. Figure 1 plots out current emotion scores by the four conditions.

**Table 1.** Means, SD of Posttest Current Feelings of Emotion Felt Towards Mother, by Condition.

	Mother appraisal down (*n =* 75)		Null (*n* = 76)		Teacher appraisal down (*n* = 76)		Mother appraisal up (*n* = 74)	
	*M*	*SD*	*M*	*SD*	*M*	*SD*	*M*	*SD*
Happiness	3.96	2.14	4.58	1.58	4.71*	1.70	5.01*	1.23
Interest	3.76	2.09	4.32	1.64	4.47	1.72	4.81*	1.36
Sadness	2.55	2.18	2.22	1.83	1.79	1.74	2.08	1.95
Anger	1.84	1.93	1.61	1.47	1.42	1.68	1.27	1.62

*Note.* *Indicates a statistically significant difference from Mother Appraisal Down condition, at *α* = .05 using Tukey HSD post hoc analysis.

#### Current Happiness

An ANOVA with the four conditions as the independent variable and posttest current strength of feelings of happiness towards the mother revealed an overall effect for condition, *F* (3, 297) = 5.08, *p* = .002, η_p_^2^ = .049. As illustrated in Table 2, in Tukey HSD post hoc analysis, the Mother Appraisal Up condition yielded higher reports of current happiness felt towards their mother compared to the Mother Appraisal Down (*p* = .001), and the teacher Appraisal Down condition (*p* = .035). Current appraisal of mother significantly correlated positively with current happiness towards mother, *r* = .774, *p* < .001, *n* = 298. The relationship between appraisal conditions and current happiness towards mother is shown in Supplemental Figure S1.

**Table 2.** Means, SD of Posttest Reports of Remembered Past Emotion Felt Towards Mother During Childhood, by Condition.

Childhood mean memory emotion	Mother appraisal down (*n =* 74)		Null (*n* = 75)		Teacher appraisal down (*n* = 75)		Mother appraisal up (*n* = 74)	
	*M*	*SD*	*M*	*SD*	*M*	*SD*	*M*	*SD*
Memory of Happiness	3.92	1.68	4.14	1.46	4.37	1.34	4.56*	1.08
Memory of Interest	3.79	1.59	3.90	1.33	4.16	1.47	4.22	1.14
Memory of Sadness	2.27	1.83	1.95	1.49	1.97	1.65	1.85	1.76
Memory of Anger	2.39	1.68	2.12	1.40	2.12	1.64	1.98	1.61

*Note.* *Indicates a statistically significant difference from Mother Appraisal Down condition, at *α* = .05 using Tukey HSD post hoc analysis.

#### Current Interest

An ANOVA with the four conditions as the independent variable and posttest current strength of feelings of interest towards the mother revealed an overall effect for condition, *F* (3, 297) = 4.84, *p* = .003, η_p_^2^ = .047. In Tukey HSD post hoc analysis, the Mother Appraisal Up condition yielded higher reports of current interest felt towards their mother compared to the Mother Appraisal Down (*p* = .001). Current appraisal of mother significantly correlated positively with current interest felt towards mother, *r* = .705, *p* < .001, *n* = 298. The relationship between appraisal conditions and current interest is shown in Supplemental Figure S2.

#### Current Sadness

An ANOVA with the four conditions as the independent variable and posttest current strength of feelings of interest towards the mother revealed condition was not a statistically significant predictor, *F* (3, 297) = 2.01, *p* = .113, η_p_^2^ = .020. There were no significant differences between groups using a post hoc Tukey HSD adjusted tests. Current appraisal of mother significantly correlated negatively with current sadness felt towards mother, *r* = −.424, *p* < .001, *n* = 298. The relationship between appraisal conditions and current happiness towards mother is shown in Supplemental Figure S3.

#### Current Anger

An ANOVA with the four conditions as the independent variable and posttest current strength of feelings of interest towards the mother revealed condition was not a statistically significant predictor, *F* (3, 297) = 1.60, *p* = .191, η_p_^2^ = .016. There were no significant differences between groups using a post hoc Tukey HSD adjusted tests. Current appraisal of mother significantly correlated negatively with current anger towards mother, *r* = −.440, *p* < .001, *n* = 298. The relationship between appraisal conditions and current happiness towards mother is shown in Supplemental Figure S4.

#### Persistence of the Effect: Current Emotions Two Weeks and Four Weeks Later

As shown in the ANOVAs in Supplemental Material Table S2 the difference between the conditions on current emotions remained similar at two weeks and four weeks after the experiment. At four weeks, the effect of condition was significant for all four current emotions (happiness, interest, sadness, anger). The patterns of the differences after four weeks are shown in Supplemental Materials Figure S5 for memory of positive emotions, and Figure S6 for memory of negative emotions.

### Hypothesis 3: Memory of Emotions

**Figure 2.**
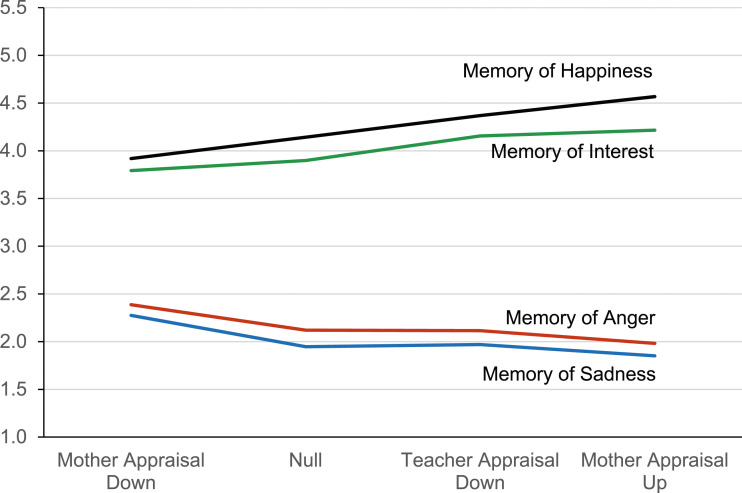
Memory of emotions towards mother in childhood (mean of grade 1, 6, and 9) in various experimental groups in Experiment 1. Notice the slope of the lines is generally in the expected direction, but is a more subtle and smaller effect compared to current emotions (compare to Figure 1).

Memory of happiness towards the mother at first, sixth, and ninth grade were strongly correlated together (*r =* .730; *r =* .602, r = .808; *p*s < .001) so to avoid multicollinearity we created a composite variable averaging these three within subject measures (see Table S1 for intercorrelations between all the memory of emotion items that comprise our DVs). Figure 2 plots the means scores of these composite measures—memory of happiness, interest, sadness, and anger by condition. Table 2 displays the means and standard deviations of these composite memory of emotion measures by emotion type and experimental condition.

#### Childhood Memory of Happiness

Using this composite measure of childhood memory of happiness towards mother as the DV, we performed an ANOVA with the four appraisal groups as the IV. The ANOVA revealed a main effect for appraisal condition, *F* (3, 294) = 2.93, *p* = .034, η_p_^2^ = .029. A Tukey HSD post hoc analysis revealed the Mother Appraisal Up condition had significantly higher memory of happiness towards mothers than the Mother Appraisal Down condition, *M*_difference_ = .649, *p* = .028 (*M, SD, n* are given in Table 2).

##### Examining the Relative Contribution of Appraisal Versus Mood

A linear regression was done with mean memory of happiness felt towards the mother in childhood as the outcome measure, and with predictor variables set as current appraisals of mother, positive mood and negative mood (PANAS affect subscales). Current appraisals of mother was the strongest predictor of memory of happiness (β = .531, *p* < .001), followed by positive mood (β = .207, *p* < .001) and negative mood (β = −.085, *p* = .071).

#### Childhood Memory of Interest

We created a composite variable averaging memory of interest in first, sixth, and ninth grade. Using this composite measure of childhood memory of interest towards mother as the DV, we performed an ANOVA with the four appraisal groups as the IV. The ANOVA revealed no significant main effect for appraisal condition, *F* (3, 294) = 1.57, *p* = .198, η_p_^2^ = .016. A Tukey HSD post hoc analysis revealed no significant differences between groups.

##### Examining the Relative Contribution of Appraisal Versus Mood

A linear regression with mean childhood memory of interest as the outcome measure found that current appraisal of mother was the strongest predictor of memory of interest (β = .406, *p* < .001), followed by positive mood (β = .288, *p* < .001) and negative mood (β = −.077, *p* = .125).

#### Childhood Memory of Sadness

Using a composite measure of childhood memory of sadness towards mother as the DV, we performed an ANOVA with the four appraisal groups as the IV. The ANOVA revealed no significant main effect for appraisal condition, *F* (3, 294) = 0.88, *p* = .453, η_p_^2^ = .009. A Tukey HSD post hoc analysis revealed no significant differences between groups.

##### Examining the Relative Contribution of Appraisal Versus Mood

A linear regression with mean memory of sadness as the outcome measure found that current appraisal of mother was the strongest predictor of memory of sadness (β = −.256, *p* < .001), followed by negative mood (β = .207, *p* < .001) and positive mood (β = −.028, *p* = .613).

#### Childhood Memory of Anger

Using a composite measure of childhood memory of anger towards mother as the DV, we performed an ANOVA with the four appraisal groups as the IV. Table 2 displays the means and standard deviations of childhood memory of interest by condition. The ANOVA revealed no significant main effect for appraisal condition, *F* (3, 294) = 0.85, *p* = .466, η_p_^2^ = .009. Tukey HSD post hoc analysis revealed no significant differences between groups.

##### Examining the Relative Contribution of Appraisal Versus Mood

A linear regression with mean memory of sadness as the outcome measure, found that current appraisal of mother was the strongest predictor of memory of sadness (β = −.274, *p* < .001), followed by negative mood (β = .200, *p* = .001) and positive mood (β = −.016, *p* = .777).

#### Fading of the Effect: Memory of Emotions Two Weeks and Four Weeks Later

As shown in the ANOVAs in Supplemental Material Table S3 the difference between the conditions on memory of emotions began to fade at two weeks and became non-significant at four weeks after the experiment. At four weeks, the effect of condition was no longer significant for all four memories of emotions (memory happiness, interest, sadness, anger).

## Experiment 1 Discussion

A pattern of results emerged in Experiment 1 that was suggestive the reappraising mothers may have an effect on current emotions towards the mother that can persist as long as 4 weeks. This was particularly strong for the positive current emotions of happiness and interest. It is puzzling why current negative emotions of anger and sadness were not significantly different between appraisal conditions immediately after the experiment, but were significantly different four weeks later. This pattern raises the possibility that time might consolidate reappraisal, but that needs further research and replication before this is confirmed.

Reappraising mothers had a weaker effect on *memories* of childhood emotions, as one might predict from the theory laid out in Patihis, Cruz et al. (2019); Figure 1 in that paper predicts a smaller effect on memories of emotion, compared to current emotions). Immediately after the experiment, and also 2 weeks later, memory of happiness in childhood towards the mother was significantly different by appraisal group. Four weeks after the experiment, these effects had faded to become non-significant. Although appraisal condition did not show significant differences for memory of interest, sadness, and anger, all these measures were associated with the continuous variable current appraisals of mother. This pattern of association does not establish cause, but it does raise the possibility of a small effect that might be revealed in future research (with either a larger manipulation, sample size, or both). The relatively small effect of appraisals on memories of emotion in research settings (the size of reappraisal might be larger in some psychotherapy settings) is consistent with comments made on this issue in Levine (1997) and Patihis, Cruz et al. (2019).

## Experiment 2

In this experiment, we utilized a different AMT sample from Experiment 1 and collected pre-test measures on current emotions and memories of emotion towards the participant’s mother (happiness, interest, sadness, anger; see Appendix S3). Eight weeks later we invited those participants back for an experiment utilizing just two conditions from the previous experiment: Mother Appraisal Up and Mother Appraisal Down. Eight weeks after the experiment, we again invited participants to participate again to assess the duration of the effect. In this experiment, we use this new design to retest Hypotheses 1 through 5.

## Experiment 2 Method

### Participants

Participants were from a US AMT sample (same sample as Exp 2 in Patihis et al., 2019) *N*_*pretest*_ = 302) of mean age 36.1 (*SD* = 11.1; range 19–70), with 77.8% (*n* = 235) female, 21.9% (66) male, and .3% (1) specifying “nonbinary.” The sample size of 300 was chosen so that the size of the effect detectable was small (G*Power calculation with *r =* .15; α = .05, Power = .8, 4 group between factors, three repeated measures ANOVA; yields a Sample size suggestion of 324; Faul et al., 2007), and so that the sample size was not so small as to lead to type 1 errors and exaggerated sample size estimates. Ethnicity was self-reported only in the second session (*N*_*experiment*_ = 202) as: 6.4% (13) Hispanic or Latino, with 93.6% (189) not Hispanic or Latino. Race was distributed as: 86.6% (175) White, 9.9% (20) Black or African American, 4.5% (9) Asian, 2.0% (4) American Indian or Alaska Native, and 1.5% (3) chose other. Mean self-reported socioeconomic status (SES), on a scale from 1 to 10, was 5.09 (*SD* = 1.6).

### Design

The design of Experiment 2 was a pretest-posttest between groups experiment. The independent variable was the participant’s current cognitive appraisal of their mother. In other words, we measured the dependent variables (memories of emotion) before and after the experiment. The pretest measures were taken 8 weeks before the experiment, and the posttest measure taken immediately after the experiment.

### Materials

#### Manipulation: Experimental Writing Prompts

The same writing prompts from Experiment 1 were used for the Mother Appraisal Up condition and the Mother Appraisal Down condition (for materials see Appendix A). This manipulation in Experiment 2 was used at the beginning of Session 2 only.

#### Manipulation Check: Current Appraisals of Mothers

The same five items used in Experiment 1 were used to assess the participants’ current appraisal of their mother on goal-relevant attributes (materials in Appendix B). The internal reliability of these current appraisal items was high (α = .921).

#### Memories of Emotion and Current Emotion

The same measures from Experiment 1 were used in Experiment 2 (shown in Appendix C). These items asked about the strength of the memory of (or current feelings of) happiness, interest, sadness, and anger.

#### PANAS

The PANAS was used as in Experiment 1. In the current experiment, both subscales had high internal reliability (Experiment 2: time 1: positive affect α = .914; negative affect α = .898) and the two subscales were negatively correlated with a small effect size, *r* = −.129, *p* = .025.

### Procedure

#### Session 1: Pretest

The procedure for Session 1 involved first presenting demographic questions. All participants completed questions assessing the current appraisal of their mothers (Appendix B). Then participants completed current emotions and memory of emotion questions (Appendix C), and then the PANAS. After completing Session 1 participants were automatically given a secret code for payment of $.50 on AMT. Session 1 took approximately 12 minutes.

#### Session 2: Experiment

Eight weeks later the same participants were invited back, via emails sent out by CloudResearch.com (formally known as TurkPrime.com; Litman et al., 2017), and 202 of the original 302 participated. Session 2 contained similar materials as Session 1 except after demographic questions, participants were randomly assigning into one of two conditions: Mother Appraisal Up or Mother Appraisal Down (for materials see Appendix A). After this experimental assignment, the participants received current appraisal questions, followed by the memory of emotion and current emotion questions. An additional subscale was added to ask, “Remember back to the day you completed Part 1 of this study,” and an example of one of the 4 items in this subscale was “When you completed Part 1 of this study, how strong was your happiness toward your mother?” Participants then completed the PANAS. After completing the session participants read a debriefing sheet and were automatically given a code for payment of $2 on AMT. The session took approximately 22 minutes.

#### Session 3: Eight weeks after the Experiment

Eight weeks after the experiment (16 weeks after the pretest), the same participants were invited back for a payment of $4 on the AMT. They were then asked similar questions to Sessions 1 and 2 (current appraisals, memory of emotion, current emotion, PANAS). Unlike Session 2, no experimental manipulation took place in Session 3.

## Experiment 2 Results

### Hypothesis 1: Manipulation Check

The manipulation did successfully affect the IV. No differences on current appraisals were found at pretest (Session 1). After the experiment in Session 2, the Mother Appraisal Up condition scored significantly higher (*M* = 3.83, *SD* = 1.12) on current appraisals of mothers than the Mother Appraisal Down condition (*M* = 3.02, *SD* = 1.30), *t* (169) = 4.39, *p* < .001, *d* = .669. Thus, the experimentally assigned writing prompts did significantly affect appraisals (the IV) as required, but with a lower effect size than Experiment 1.

### Hypothesis 2: Current Emotions

At pretest (Session 1), no significant differences between conditions were found on current emotions (happiness, interest, sadness, anger towards mother; *p*s > .455).

In Session 2, immediately after the experiment, current happiness towards the mother was significantly higher in the Mother Appraisal Up condition (*M* = 4.45, *SD* = 1.66) compared to the Mother Appraisal Down condition (*M* = 3.78, *SD* = 2.10), *t* (169) = 2.32, *p* = .022, Cohen’s *d* = .354. Current interest towards the mother was marginally higher in the Mother Appraisal Up condition (*M* = 4.42, *SD* = 1.64) compared to the Mother Appraisal Down condition (*M* = 3.88, *SD* = 1.93), *t* (169) = 1.96, *p* = .051, *d* = .301. Current sadness and anger were not significantly different between the conditions (*p =* .779 and .359 respectively).

In a series of mixed linear models with current emotion as the dependent variable, with Session (1, 2) as a within subject variable, and Condition (Appraisal Up, Appraisal Down) as the between-subjects variable. There was a significant interaction (Session × Condition) when current happiness was the dependent variable (*p =* .032). The Session × Condition interaction was not significant (marginal) for current interest (*p* = .066), and not significant for current sadness (*p* = .963) and anger (*p* = .248).

In Session 3 eight weeks after the experiment, the differences between the appraisal conditions had faded to become non-significant on current emotions towards the participant’s mother (happiness, interest, sadness, anger; *p*s > .130).

### Hypothesis 3: Memory of Emotion

In the Session 1 pretest, as we would expect no difference between the appraisal groups was found on memory of childhood emotions towards the participant’s mother (happiness, interest, sadness, anger; *p*s > .221).

In Session 2 after the experiment, memory of happiness was not significantly higher in the Appraisal Up condition (*M* = 4.22, *SD* = 1.34) compared to the Appraisal Down condition (*M* = 4.22, *SD* = 1.34), *t* (201) = 1.83, *p* = .069. There were no significant differences on memory of interest, sadness, and anger (*p* > .238).

A series of mixed linear models were conducted with memories of emotion as the dependent variable, with Session (1, 2) as a within subject variable, and Condition (Mother Appraisal Up, Mother Appraisal Down) as the between-subjects variable. The Session × Condition interaction was not significant for memory of happiness (*p* = .336), memory of interest (*p* = .772), memory of sadness (*p* = .573), and memory of anger (*p* = .489).

In a series of mixed model with current emotion at pretest (Session 1) and memories of that same emotion (Session 2) as the dependent variable, with Session (1, 2) as the within-subject variable, and Condition (Mother Appraisal Up, Mother Appraisal Down) as the between-subjects variable. The Session × Condition interaction was not significant for memory of happiness (current at pretest; *p* = .329), memory of interest (*p* = .338), memory of sadness (*p* = .815), and memory of anger (*p* = .540).

In Session 3, eight weeks after the experiment, there were no differences between the conditions on memory of happiness, interest, sadness, or anger (*p*s > .125).

## Discussion of Experiment 2

In Experiment 2, we found the participants’ current happiness towards mother did go down in the appraisal down condition, and there appeared to be a small effect for memory of happiness from childhood. There was a marginal result for current interest. For the negative emotions, though, there were no significant differences in either the current emotion analysis, or the memories of emotion analysis.

With the introduction of the pretest measures of current emotion and memories of emotion (Experiment 2), the malleability of these constructs appears reduced (Experiment 1). We might speculate that the process of answering the pretest emotion and memory of emotion questions anchors the participants’ responses. This anchoring makes it harder to move these constructs when participants subsequently answer the same question after the experiment. This is consistent with the relatively lower effect sizes that we found in Patihis, Cruz et al. (2019) in the pretest/posttest design (Experiment 2), compared to the experiment-posttest design (Experiment 1). Since the malleability of memories of emotion is a difficult effect to experimentally manipulate, the pretest design may not be as fruitful as purely posttest designs in future research.

## General Discussion

In two experiments we found support for the idea that non-suggestive writing prompts that simply ask participants to write a few sentences about recent examples of their mother’s behavior is sufficient to change current appraisals of those mothers. In Experiment 1 we found that current positive emotions felt towards mothers, such as happiness, to be more malleable than negative emotions, such as anger and sadness. Current appraisals were associated with memories of emotion in general, though this does not show causality in each emotion. We found in an experiment with a posttest design that manipulating current appraisals did lead to changes in memory of happiness towards mothers in childhood. Perhaps surprisingly, four weeks after the experiment, there was a persistence in differences on current emotions by appraisal group (all emotions showed significant differences by condition at four weeks, perhaps surprisingly), but by four weeks all memory of emotion effects had faded. In Experiment 2 we found the pretest design tended to lessen the effects compared to Experiment 1. In Experiment 2 the current happiness felt toward mothers was significantly different by group, but many other effects were not significant.

### Theory Implications

Cognitive appraisal theories of emotion, and reconstructive memory theories, are somewhat supported by the evidence. Reappraisals of mothers had an effect on current emotions, especially positive emotions (happiness, interest). This experimental approach provides some support for the cognitive appraisal model of emotions that posits that current appraisals are a major cause of emotions (Lazarus, 1991). Because Freud’s theory of catharsis (see Breuer & Freud, 1895/1953) and LeDoux’s (1992) indelibility theory would predict accurate memories of emotions, our research and other memory of emotion research (e.g., Chang & Overall, 2024; Levine et al., 2021) do not support catharsis and indelibility theories. In the posttest-only experiment we found some evidence of the effect on memories of positive emotions, and this supports a mix of reconstructive memory and appraisal theories put forward by Levine (1997) and Patihis, Cruz et al. (2019). This theory posits that reappraisals can change memory of emotions (to a lesser extent than current emotions, see Figure 1 in Patihis, Cruz et al., 2019).

### Practical Applications

The fact that non-suggestive writing prompts can change current appraisals of a parent perhaps illustrates the iatrogenic risks that such processes may elicit in psychotherapy if such methods are done a lot. Even with good therapists that do not use suggestions, reappraisals may occur inadvertently. Our prompts in the appraisal down condition simply asked for recent real examples of when the mother had shown a lack of an attribute (e.g., generosity). So, the process of reporting true recent examples, as happens often in talk therapy, can diminish current appraisals of a parent. Perhaps psychotherapies should inform clients that one of the iatrogenic risks of discussing parents (or indeed other relationships, such as partners) can be the lowering of current appraisals. In the past, the typical guidelines from memory distortion researchers to therapists was that if one simply avoids false suggestions, the risks of memory distortions are low. Now, with this research (as well as Patihis, Cruz et al., 2019) we might also add that even truthful discussion of past behaviors of a parent may reduce current appraisals of the parent—perhaps to the detriment of the client’s life.

Current emotions towards a parent are important, as are memories of happiness once felt in childhood. It is important that therapists everywhere learn about these processes, so they know it is easy to change emotions and memories even in non-suggestive discussion of parent behavior or character. Indeed, it may be a good idea to measure current emotions towards parents and partners before and after therapy to help identify iatrogenic harm. For more than a hundred years (since the 1880s French schools of hypnosis, followed in the 1890s by Freud; see Patihis & Younes, 2015), therapists have been encouraging clients to talk about their parents without measuring the possible side effects of lowered evaluation of parents, worsened current emotions towards parents, and contaminated memories of childhood emotions towards parents.

### Relation to Past Research

Compared to Patihis, Cruz et al. (2019) results for the memories of love, the current results for memories of happiness, interest, sadness, and anger were generally less. This may because the writing prompts (common to both) were written with the emotion love in mind—in other words they were designed to elicit changes in appraisals in those attributes that would most affect feelings of love. In future research, if they were rewritten to change appraisals in attributes that might facilitate or diminish anger (for example), then the effect on memories of anger might vary more between conditions. It is interesting to notice that in the current experiments that happiness and memory of happiness was most malleable to appraisal changes, and this is a positive emotion (like love). It makes some logical sense that raising appraisals of attributes like generosity of mothers would raise memories of happiness, perhaps moreso than lowering appraisals of generosity would raise memories of anger or sadness. It may be simply an artifact of the experiments that memory of positive emotions were more malleable than memory of negative emotions.

These findings are also complementary to other past research that has tended to find subtle changes in memory of emotion. This includes longitudinal research that has found memories of emotions are malleable in relation to a number of events (e.g., presidential races (Levine, 1997), political events (Levine et al., 2021), legal cases (Levine et al., 2001), and disasters (Levine et al., 2005). Past experiments are also somewhat in keeping with our current experimental results that the valence of the appraisal will bias the memory for emotions. For example, Keuler and Safer (1998) found that memory for pre-exam anxiety was malleable following on the positive reappraisal of success compared to no reappraisal (see also Safer et al., 2002). Similarly, Chang and Overall (2024) found that cognitive reappraisal task was associated with biasing memories of depressive symptoms during Covid lockdown. In general, past research is congruent with our findings.

### Limitations and Future Research

One of the main limitations of the current experiments is the relatively small effects in Experiment 2, and thus a lack of vigorous replication that would be ideal. This was likely due to the pretest measures of the DV that may have in effect anchored the posttest measures and made them resistant to change. To be specific, this may occur when participants remember how they previously answered, and then fixing their answers at—or close to—that same rating scale response. For this reason, future research might avoid pretest measures of these DVs to detect the subtle effects that small reappraisals may cause on memory of emotions. Alternatively, power could be boosted by increasing the reappraisal manipulation (if ethical to do so) or increasing sample sizes. Reappraisals in laboratory or online experiments will always likely be small, so future research could investigate the more powerful reappraisals in psychotherapy that tends to reappraise parents (or partners) over many sessions (e.g., psychodynamic therapy or trauma-informed therapy focused on childhood). These reappraisals in naturally occurring setting would have occurred with or without the research measuring emotions and memories of emotion afterwards, so it would be ethically justifiable. Indeed, if therapies that discuss parents and partners do lower positive emotions or memories of positive emotions, discovering this iatrogenic side-effect in research is important to discovery. Another limitation is generalizability, for example to other target-people such as fathers or partners, and this can be examined in future research. Notably though, the current study shows that Patihis, Cruz et al. (2019) does generalize to other emotions and memories of emotion.

## Conclusion

There are several findings that are important for psychotherapists, psychologists, and the general public to know about. First, even using *non*-suggestive prompts to ask people to give examples of when their parent were generous (or not) can change people’s current appraisals of that parent. In real life situations and therapy, these reappraisals will likely be larger than short lab-tasks can induce. Second, this can in turn change their current emotions towards that parent. In therapies that reappraise parents or partners much more than brief lab tasks, this effect might be larger. And third, these reappraisals can also affect memories of childhood happiness that the participant remembers feeling towards their mother. This experimental research points to the need for appraisals of parents (and/or spouses/partners), emotions, and memories of emotions, to be measured before and after therapy to investigate inadvertent (and well-meaning) iatrogenic harm to relationships.

## Supplemental Material

Supplemental Material - Reappraising a Parent can Occur With Non-Suggestive Questions: Changing Emotions and Memories of Emotion Supplemental Material for Reappraising a Parent can Occur With Non-Suggestive Questions: Changing Emotions and Memories of Emotion by Lawrence Patihis, and Mario E. Herrera in Psychological Reports.

Supplemental Material - Reappraising a Parent can Occur With Non-Suggestive Questions: Changing Emotions and Memories of Emotion Supplemental Material for Reappraising a Parent can Occur With Non-Suggestive Questions: Changing Emotions and Memories of Emotion by Lawrence Patihis, and Mario E. Herrera in Psychological Reports.

## Data Availability

For Patihis & Herrera (in press 2024) Reappraising a Parent can Occur With Non-suggestive Questions: Changing Emotions and Memories of Emotion. The datasets for both experiments can be found here: https://osf.io/rhfuw/.
