# Supplementary material for: Reappraising a Parent can Occur With Non-suggestive Questions: Changing Emotions and Memories of Emotion
Source: Psychol Rep. 2024 Sep 11;129(4):3215–36. doi: 10.1177/00332941241283413 (PMC13287537; doi:10.1177/00332941241283413)
Supplement: Supplemental Material - Reappraising a Parent can Occur With Non-Suggestive Questions: Changing Emotions and Memories of Emotion [file sj-pdf-1-prx-10.1177_00332941241283413.pdf]

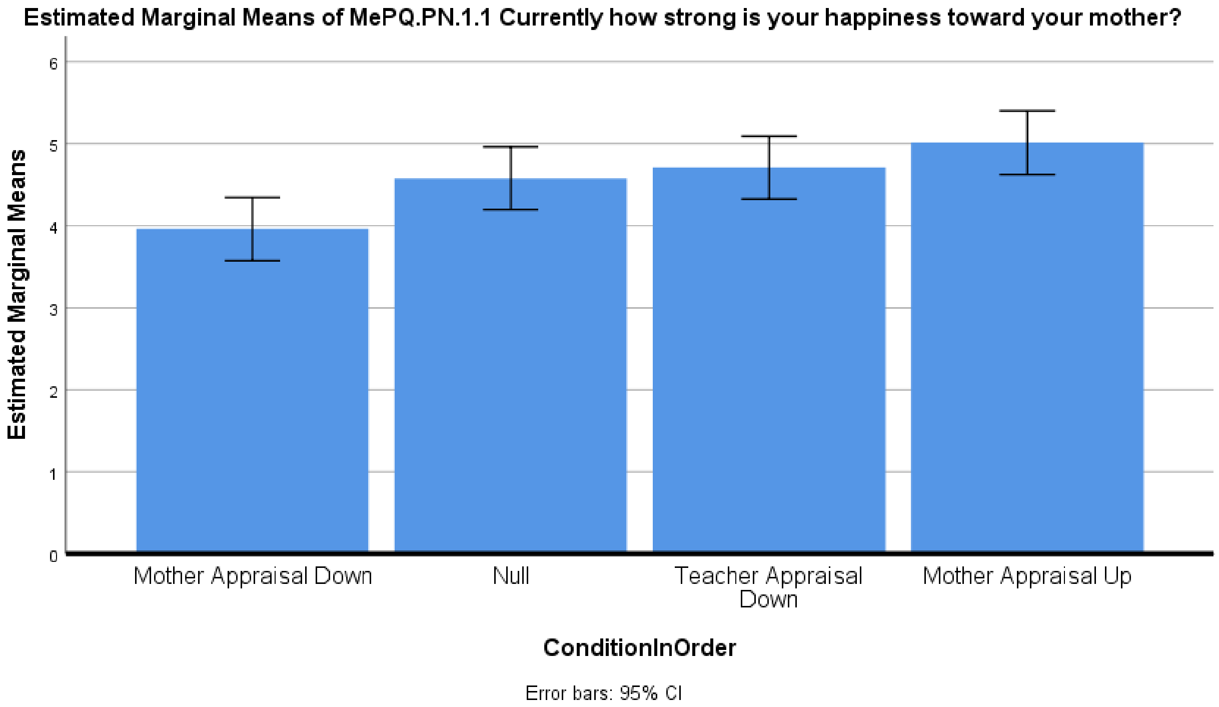

Figure S1. Experiment 1 appraisal group comparisons on current happiness, with 95% confidence intervals as error bars.

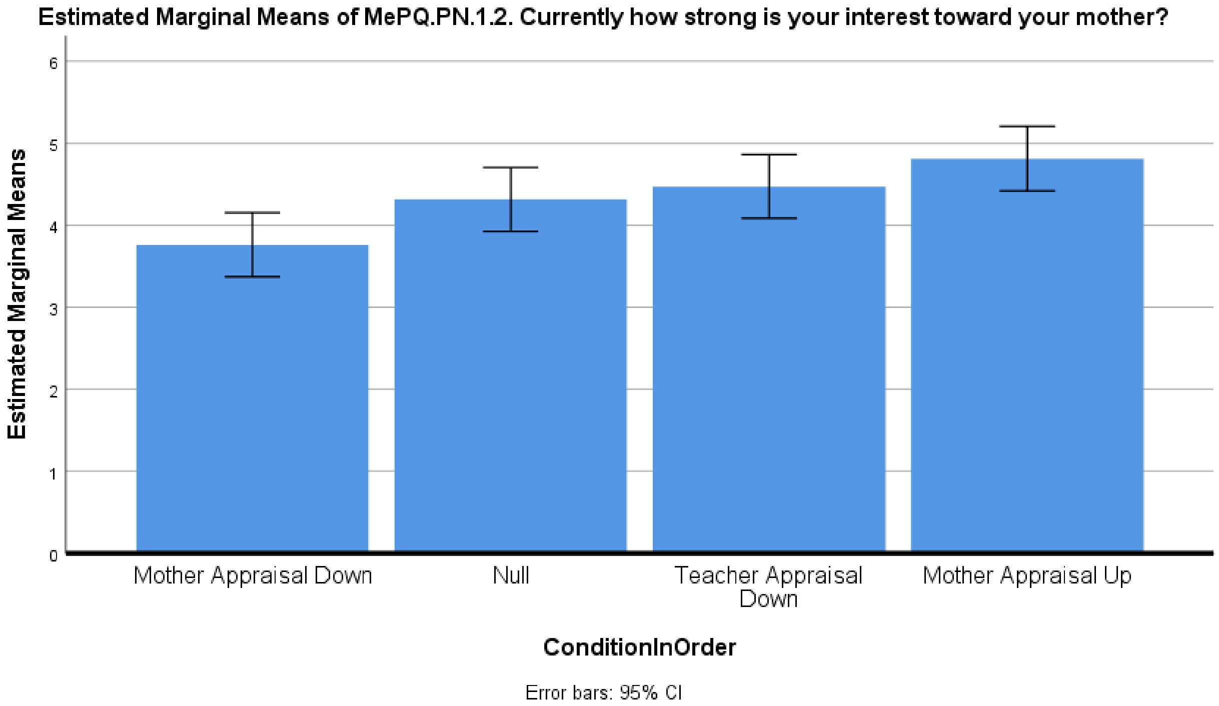

Figure S2. Experiment 1 appraisal group comparisons on current interest, with 95% confidence intervals as error bars.

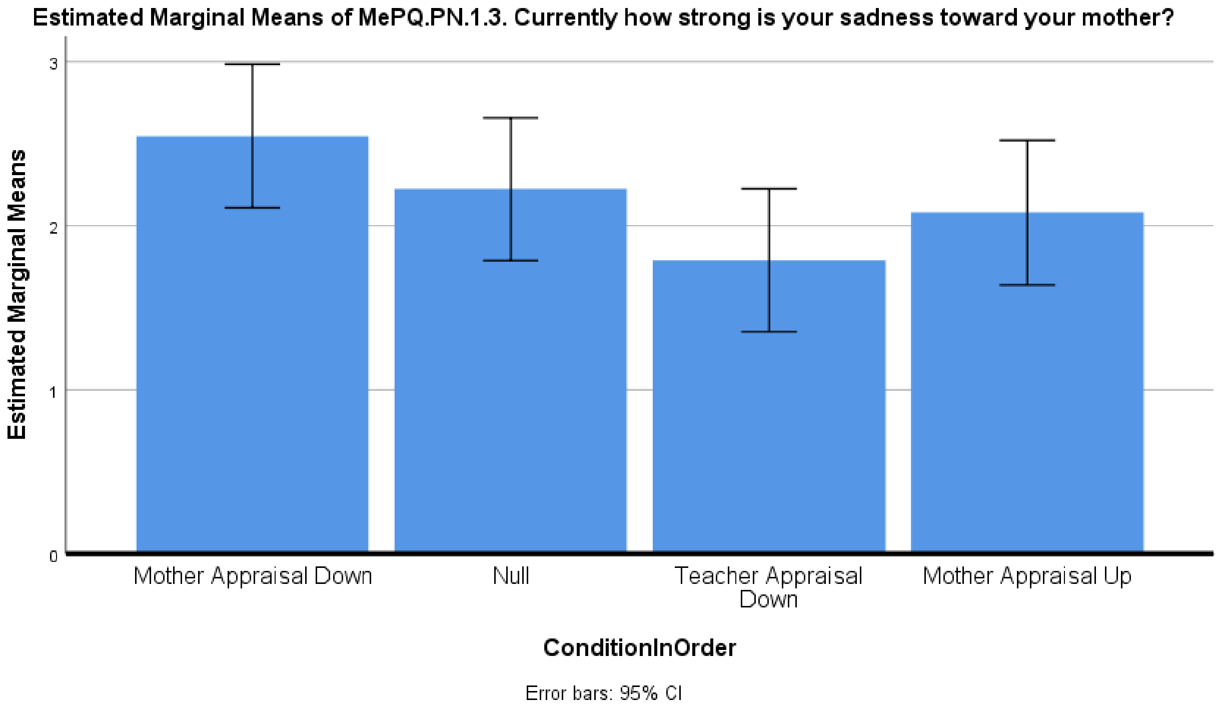

Figure S3. Experiment 1 appraisal group comparisons on current sadness towards mother, with 95% confidence intervals as error bars.

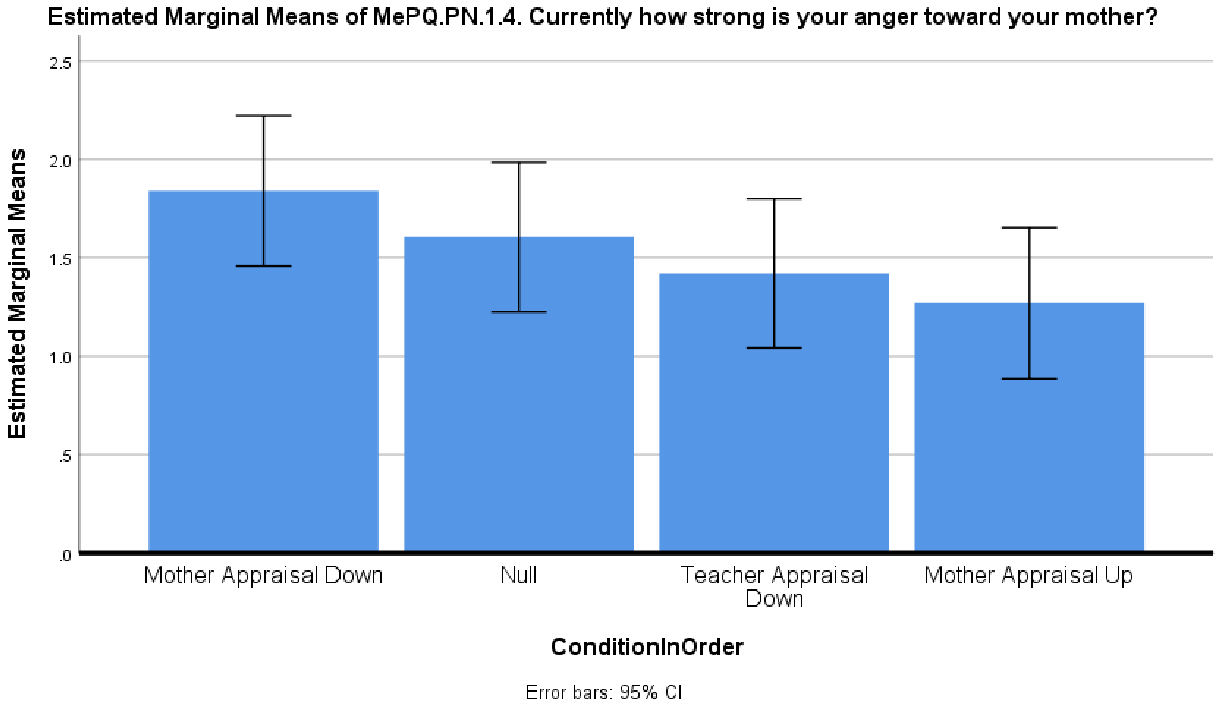

Figure S4. Experiment 1 appraisal group comparisons on current anger towards mother, with 95% confidence intervals as error bars.

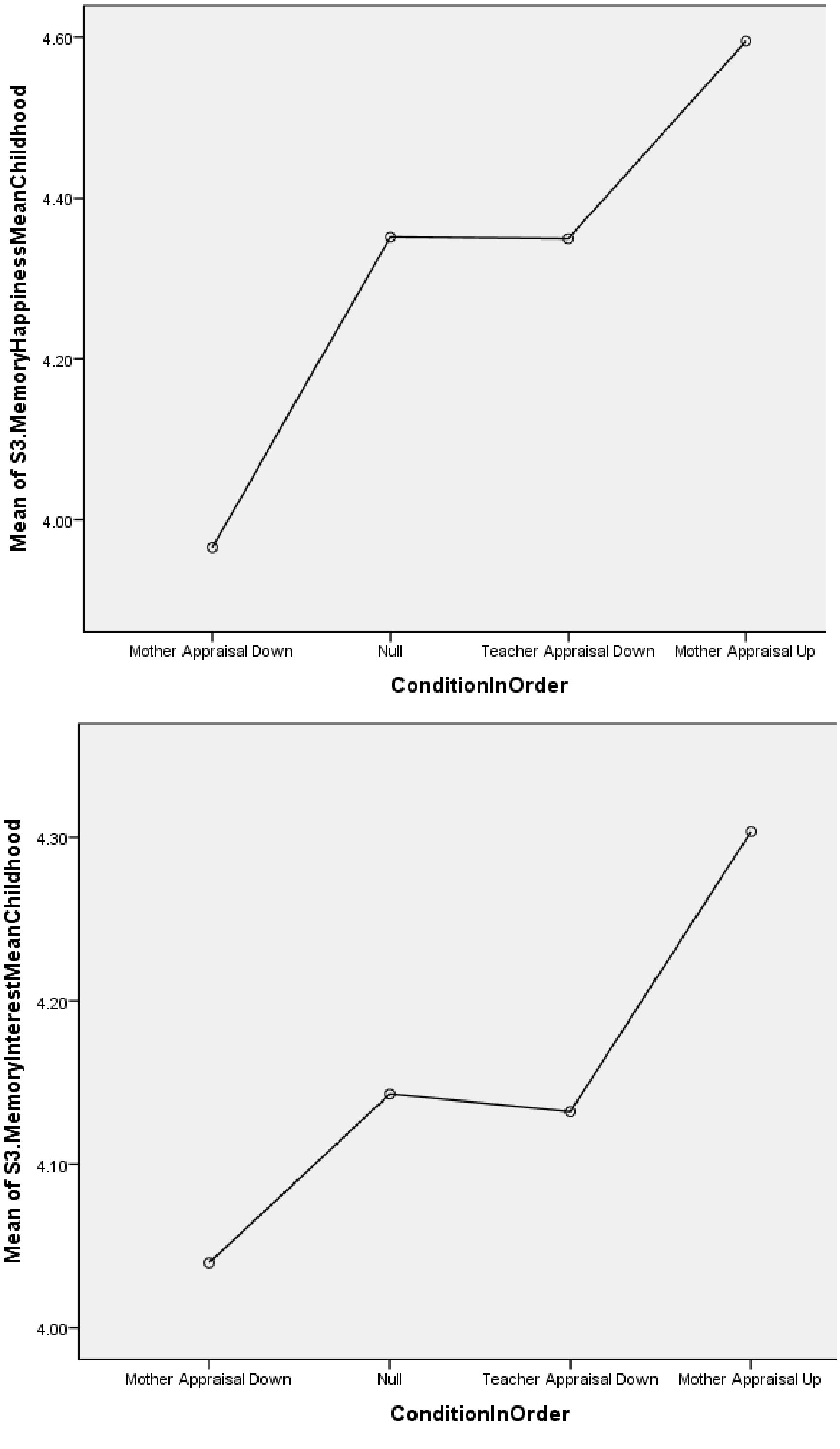

Figure S5. Experiment 1, Session 3 (four weeks after experiment): memory of positive childhood emotions towards participant's mother by condition in Experiment 1 at four weeks after the experiment. Top graph shows memory of happiness, bottom graph shows memory of interest.

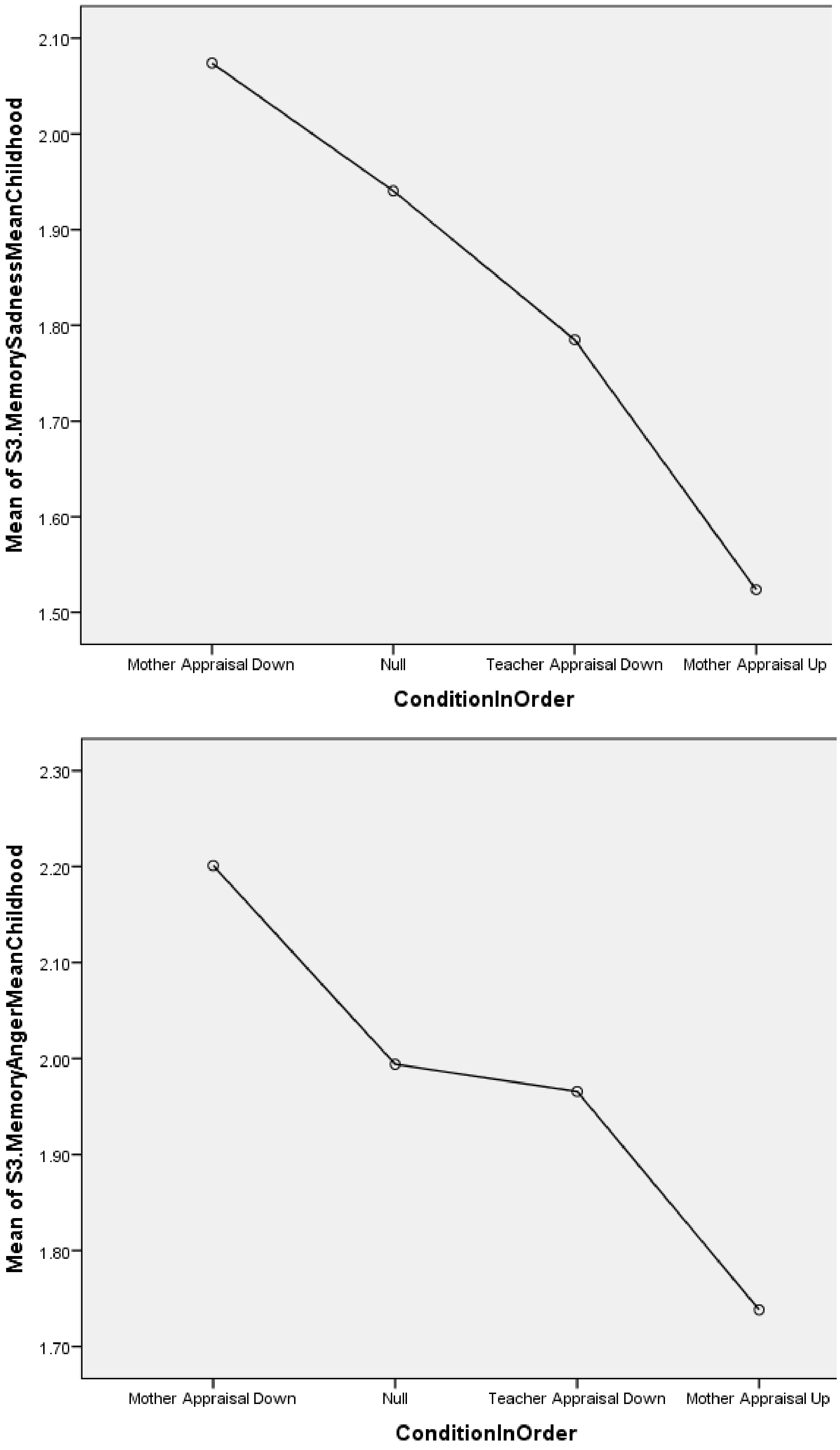

Figure S6. Memory of negative childhood emotions towards participant's mother by condition in Experiment 1 at four weeks (Session 3) after the experiment. Top graph shows memory of sadness, bottom graph shows memory of anger.

Table S1

Spearman's Rho Correlations between Items that comprise our Memory of Emotion Outcome Measures (Experiment 1 data; N *= 295)*

	G1 I	G1 S	G1A	G6 H	G6 I	G6 S	G 6A	G9 H	G9 I	G9 S	G9 A	Hap	Int	Sad	Ang
G1 H	.765**	-.439**	-.465**	.700**	.529**	-.396**	-.322**	.595**	.409**	-.371**	-.230**	.590**	.520**	-.304**	-.357**
G1 I	1.000	-.330**	-.369**	.638**	.657**	-.252**	-.244**	.563**	.485**	-.243**	-.202**	.565**	.551**	-.268**	-.358**
G1 S		1.000	.801**	-.328**	-.189**	.730**	.617**	-.239**	-.073	.596**	.404**	-.285**	-.260**	.388**	.440**
G1 A			1.000	-.355**	-.268**	.641**	.701**	-.269**	-.152**	.532**	.463**	-.277**	-.276**	.314**	.432**
G6 H				1.000	.811**	-.482**	-.475**	.796**	.636**	-.408**	-.392**	.586**	.539**	-.308**	-.348**
G6 I					1.000	-.280**	-.355**	.677**	.714**	-.246**	-.307**	.470**	.514**	-.258**	-.256**
G6 S						1.000	.792**	-.414**	-.237**	.765**	.597**	-.325**	-.311**	.436**	.470**
G6 A							1.000	-.416**	-.295**	.649**	.686**	-.275**	-.287**	.391**	.456**
G9 H								1.000	.831**	-.470**	-.531**	.551**	.503**	-.321**	-.347**
G9 I									1.000	-.257**	-.444**	.391**	.467**	-.199**	-.250**
G9 S										1.000	.760**	-.340**	-.291**	.468**	.493**
G9 A											1.000	-.322**	-.313**	.385**	.493**
Hap												1.000	.833**	-.415**	-.554**
Int													1.000	-.352**	-.506**
Sad														1.000	.671**

Note. G1 = Grade 1. G6 = Grade 6. G9 = Grade 9. I = Memory of Interest towards mother. S = Memory of Sadness. A = Memory of Anger. H = Memory of Happiness.

Hap = Current feelings of happiness towards mother. Int = Current feelings of interest towards mother. Sad = Current sadness towards mother. Ang = current anger towards mother.

Table S2. Persistence of the Effect in Experiment 1. Current Emotions Two Weeks Later (Session 2) and 4 Weeks Later (Session 3)

ANOVA Two Weeks Later:

	df	*F*	*p*
S2. Currently how strong is your happiness toward your mother?	3	6.121	.001
S2. Currently how strong is your interest toward your mother?	3	5.619	.001
S2. Currently how strong is your sadness toward your mother?	3	2.279	.080
S2. Currently how strong is your anger toward your mother?	3	3.554	.015

Note. N = 231. S2 = Session 2 (2 weeks after the experiment)

ANOVA Four Weeks Later

	df	*F*	*p*
S3. Currently how strong is your happiness toward your mother?	3	3.519	.016
S3. Currently how strong is your interest toward your mother?	3	3.817	.011
S3. Currently how strong is your sadness toward your mother?	3	3.507	.016
S3. Currently how strong is your anger toward your mother?	3	4.538	.004

Note. N = 237. S3 = Session 3 (4 weeks after the experiment)

Table S3. Persistence of the Effect in Experiment 1: Memory of Emotions towards Mother Two Weeks Later (Session 2) and 4 Weeks Later (Session 3)

Session 2 ANOVA (Two Weeks after Experiment)

	df	*F*	*p*
S2. Memory of Happiness (Mean Childhood)	3	3.962	.009
S2. Memory of Interest (Mean Childhood)	3	2.569	.055
S2. Memory of Sadness (Mean Childhood)	3	.963	.411
S2. Memory of Anger (Mean Childhood)	3	1.771	.154

Note. Mean Childhood = mean of Grade 1, Grade 6, and Grade 9 memories of emotion scores.
N = 231

Session 3 ANOVA (Four Weeks after Experiment)

	df	*F*	*p*
S3. Memory of Happiness (Mean Childhood)	3	2.217	.087
S3. Memory of Interest (Mean Childhood)	3	.372	.773
S3. Memory of Sadness (Mean Childhood)	3	1.429	.235
S3. Memory of Anger (Mean Childhood)	3	.931	.426

Note. N = 237.
