# Supplementary material for: Reappraising a Parent can Occur With Non-suggestive Questions: Changing Emotions and Memories of Emotion
Source: Psychol Rep. 2024 Sep 11;129(4):3215–36. doi: 10.1177/00332941241283413 (PMC13287537; doi:10.1177/00332941241283413)
Supplement: Supplemental Material - Reappraising a Parent can Occur With Non-Suggestive Questions: Changing Emotions and Memories of Emotion [file sj-pdf-2-prx-10.1177_00332941241283413.pdf]

Appendix A
Randomly Assigned Appraisal Condition Writing Prompts

Experimental Groups:

Mother Appraisal Up Condition

Please write 3-4 sentences giving the most recent examples of when your mother showed warmth towards you.

Please write 3-4 sentences giving the most recent examples of when your mother showed competence (effectiveness) in her life.

Please write 3-4 sentences giving the most recent examples of when your mother showed generosity towards you.

Please write 3-4 sentences giving the most recent examples of when your mother offered good guidance towards you.

Please write 3-4 sentences giving the most recent examples of when your mother gave and received love from you.

Mother Appraisal Down Condition

Please write 3-4 sentences giving the most recent examples of when your mother showed a lack of warmth towards you.

Please write 3-4 sentences giving the most recent examples of when your mother showed a lack of competence (effectiveness) in her life.

Please write 3-4 sentences giving the most recent examples of when your mother showed a lack of generosity towards you.

Please write 3-4 sentences giving the most recent examples of when your mother gave you bad guidance.

Please write 3-4 sentences giving the most recent examples of when your mother did not give love to you.

Comparison Groups:

Teacher Appraisal Down (Study 2) Conditions

The same wordings were used as the corresponding mother condition (shown above), except substituting the words "your mother" with "a teacher."

Null Condition

Participants received no writing prompts and proceeded to the next part of the study.

Note. For the writing prompt a minimum number of 50 characters was required to be entered for each prompt before the participants could proceed to the next page.

Appendix B

Current Appraisal of Mothers Questions

How do you evaluate your mother *currently* on:

[Appraisal of attributes of the mother (attributes relevant to or important in parenting): composite score = 5 item mean]

1. Current emotional warmth of your mother:
    - Poor [coded 1]
    - Fair [coded 2]
    - Good [coded 3]
    - Very Good [coded 4]
    - Excellent [coded 5]
    - Not applicable [coded as missing data]
2. Current competence (effectiveness) in life of your mother:
3. Current generosity of your mother:
4. Quality of current parenting in terms of good guidance from your mother:
5. Quality of current parenting in terms of *giving and receiving love* from your mother:

Appendix C

Memories of Emotion Item Wording (see note for current emotion wording)

[Instructions seen on previous pages:]

First Year of Elementary School

Remember back to how you felt about your mother during the year in which you were in first grade (how you felt toward her at that time).
First grade is typically experienced at ages 6-7 years in the United States, and is the first year of Elementary School.

[Instructions seen on same page as the questions given below:]

Emotions Towards Mother in First Grade

1. During the whole year when you were **in first grade**, *how strong on average* was your **happiness** toward your **mother**?

I Never Knew This Parent At All	Nonexistent 0	1	2	3	4	5	Extremely Strong 6
○	○	○	○	○	○	○	○

2. During the whole year when you were **in first grade**, *how strong on average* was your **interest** toward your **mother**?

I Never Knew This Parent At All	Nonexistent 0	1	2	3	4	5	Extremely Strong 6
○	○	○	○	○	○	○	○

3. During the whole year when you were **in first grade**, *how strong on average* was your **sadness** toward your **mother**?

I Never Knew This Parent At All	Nonexistent 0	1	2	3	4	5	Extremely Strong 6
○	○	○	○	○	○	○	○

4. During the whole year when you were **in first grade**, *how strong on average* did you feel **anger** toward your **mother**?

I Never Knew This Parent At All	Nonexistent 0	1	2	3	4	5	Extremely Strong 6
○	○	○	○	○	○	○	○

[*Notes.* In other subscales (Grade 6, 9, and Current) changed the words "first grade." An example item of the Current subscale is: "Currently *how strong is* your **happiness** toward your **mother**?" The anchor "*I never knew this parent at all*" is coded as missing data.]
